# A Brief Review on Advanced Sandwich Structures with Customized Design Core and Composite Face Sheet

**DOI:** 10.3390/polym14204267

**Published:** 2022-10-11

**Authors:** Santosh Kumar Sahu, P. S. Rama Sreekanth, S. V. Kota Reddy

**Affiliations:** School of Mechanical Engineering, VIT-AP University, Amaravati 522237, Andhra Pradesh, India

**Keywords:** multifunctional composites, sandwich structures, mechanical properties, damage mechanics, 3D printing

## Abstract

Sandwich structures are a class of multifunctional high-performance structural composites that have the advantages of being lightweight, of a high strength-to-weight ratio, and of high specific energy absorption capabilities. The creative design of the core along with the apposite material selection for the fabrication of the face sheet and core are the two prerequisites with encouraging areas for further expedition towards the fabrication of advanced composite sandwich structures. The current review work focused on different types of core designs, such as truss, foam, corrugated, honeycomb, derivative, hybrid, hollow, hierarchical, gradient, folded, and smart core along with different composite materials accessible for face sheet fabrication, including fiber-reinforced composite, metal matrix composite, and polymer matrix composite are considered. The joining method plays a major role for the performance evolution of sandwich structures, which were also investigated. Further discussions are aligned to address major challenges in the fabrication of sandwich structures and further enlighten the future direction of the advanced composite sandwich structure. Finally, the work is summarized with a brief conclusion. This review article provides wider guidelines for researchers in designing and manufacturing next-generation lightweight multilayer core sandwich structures.

## 1. Introduction

**Figure 1:**
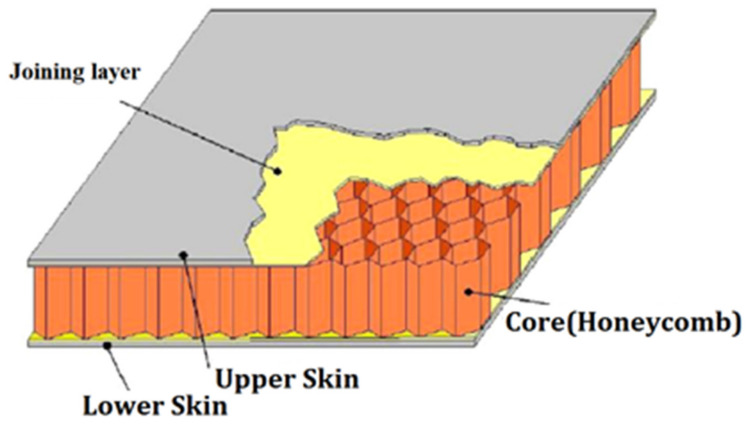
Typical sandwich structure [4].

Composites have many advantages over conventional virgin materials, such as steel and aluminum, as these provide weight reduction, fuel saving, high specific strength and stiffness, increased stability, and corrosion resistance. Due to these advantages, the use of composites is growing in areas such as aircrafts, high speed trains, space crafts, as well as the automotive, marine, and building industries [1,2]. Although various types of composites are available, sandwich structures have attracted much interest in recent years due to their unique properties, i.e., high bending resistance, high stiffness, light weight, and shock absorption capability [3]. Sandwich structure is a special form of a laminated composite formed by two stiff facings at the top and bottom (e.g., alloys of aluminum, fiber-reinforced polymer (FRP) composites and epoxy/carbon composites, etc.) along with a lightweight core (e.g., honeycomb, truss, and foam, etc.). Both the face sheet and the core were bonded by a suitable joining technique. The schematic representation of a typical sandwich beam is shown in Figure 1 [4]:

The unique advantages of sandwich structures are that they have an improved energy absorption ability, excellent ballistic resistance, and extraordinary thermal and noise isolation properties. These advantages of sandwich structures led to a wide scope of engineering applications, such as in the marine, automotive, aeronautical, and aviation industries. The first use of a sandwich composite was reported during World War II on a “Mosquito” and a “Vampire” aircraft, wherein end-grain balsa was used as the core and plywood as the skin [5]. The Korean Tilting Train eXpress body is made by sandwich structure elements consisting of carbon fabric/epoxy prepreg face sheets and an aluminum honeycomb core, which reduces the weight of the car’s upper body by 39%, while also reducing external sound, lowering wheel–rail forces, reducing ground vibrations, and enhancing weight reduction [6]. For their space shuttle orbiter, NASA uses graphite/epoxy honeycomb sandwich composites due to their low density, their minimum thermal expansion, and their higher modulus of elasticity [7]. Modern swift racing boats use honeycomb core sandwich structures in places such as decks and sax boards, aiming to furnish extra stiffness and reduce the overall weight [8]. The loudspeakers diaphragms consist of a honeycomb sandwich disk to provide a wider frequency range [9]. The smart slab technique adopted the honeycomb sandwich pixel LED so that displays can be integrated into the architecture [10]. For its car body chassis, Ferrari F50 has used carbon/epoxy face sheets (skin) embedded on Nomex honeycomb core sandwich constructions [11]. Al-Bahar Towers, Abu Dhabi, uses honeycomb structures, which open and close relative to the sun’s movement, which helps to reduce the gain by about 50% without compromising the effective transfer of natural light inside the tower [12]. It is observed that composite sandwich structures are adopted in multifarious areas.

**Figure 2:**
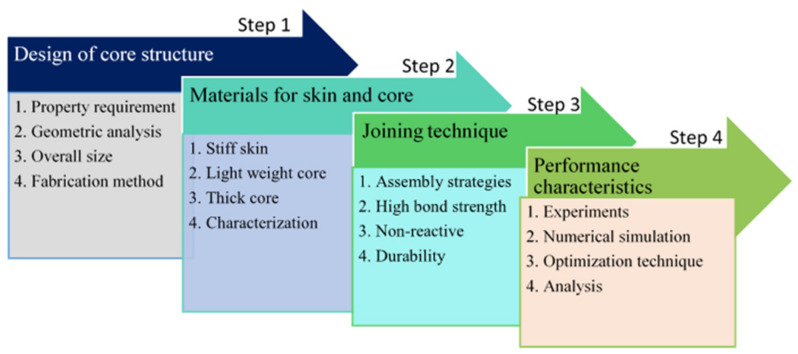
Steps to achieve advanced sandwich structure with extraordinary performance.

The performance sandwich structures were first influenced by the topological design. Secondly, by the choice material and its processing method. Thirdly, by the technique that the joining method adopted to fabricate the sandwich beam. Figure 2 shows insight into the detailed procedure for the development of an advanced composite sandwich structure. The novelty of the present review work lies in the thorough research on the different creative core structures, their material, their composite skin (face sheet) materials, and their joining techniques. The review also focuses on different testing techniques and methods to assess the performance of the advanced sandwich composite structures and the challenges thereof. The advancement in the development of new designs, materials, and fabrication techniques upgraded the sandwich structure by executing cell topology planning and optimization techniques. Considering the exhaustive picture of sandwich structure for various applications, an extensive review was carried out in the current investigation.

## 2. Design of Core Structure

**Figure 3:**
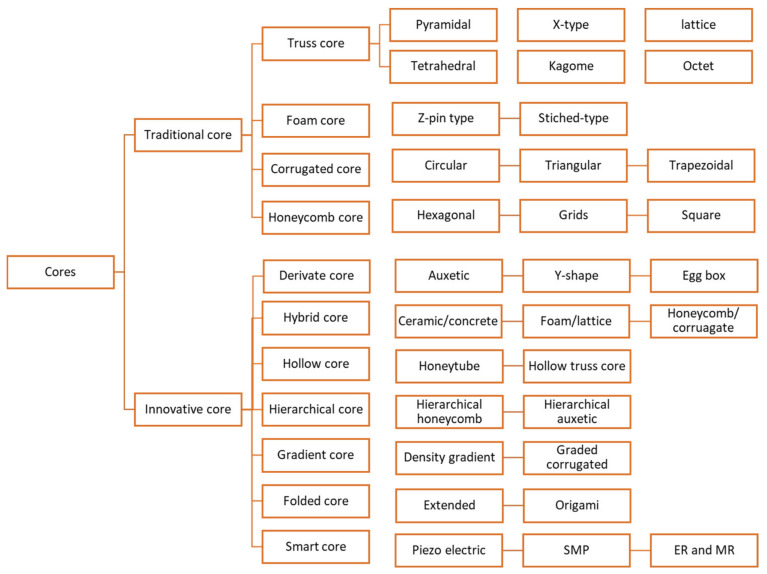
Types of cores.

The core is the centrally positioned layer of a sandwich structure. The prime requirement of the core layer is to augment the thickness of the sandwich structure without up surging the overall weight. The core provides compressive and shear strength in the sandwich structure [13]. The core design is the initial step for the creation of the sandwich structure. There are several types of cores which are broadly classified into traditional and innovative cores. Examples of traditional cores are honeycomb, foam, corrugated, and truss cores, etc. The innovative cores are derivate, hybrid, hollow, hierarchical, graded, folded, and smart cores, etc. [14]. Figure 3 shows the summarized view of different types of sandwich structures based on the types of cores. Moreover, the different types of cores are discussed in detail in the subsequent sections.

### 2.1. Traditional Core Structure

The traditional core can be broadly divided into two categories: homogeneous and non-homogeneous support of the skin. The homogeneous support cores are foam/cellular cores, whereas the non-homogeneous support cores are textile/pin/truss/pyramidal, corrugated, and honeycomb cores. In the subsequent sections, the traditional cores are discussed in detail.

#### 2.1.1. Textiles/Lattice/Pin/Truss Core

**Figure 4:**
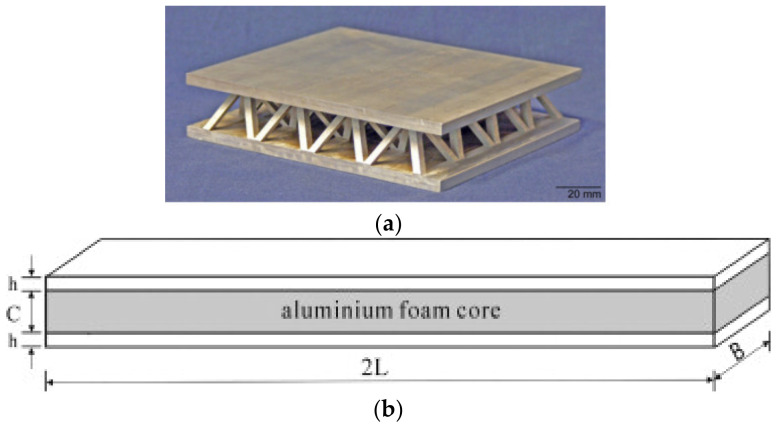
(**a**) Lattice sandwich structure [17]; (**b**) Aluminum foam core design [27].

These punctual supports with fully open cell metallic truss cores possess elevated strength and explosion resistance, as the cells are built with stainless steel tubes [15]. Lattice truss cores are used for load-bearing structures due to their high specific strength and stiffness [16]. These cores may be tetragonal, pyramidal, or Kagome patterns. Figure 4a shows the lattice core [17]. The following is the brief literature survey in the context of truss core.

Wang et al. [18] reported mechanical and failure behavior of the X-type carbon fiber lattice core with variable relative density from 5.6 to 1.8. It was observed that a core with a 5.6 relative density has higher compressive and shear stress, with a top skin debonding failure when shear force approaches the ultimate load-sustaining capacity. Mei et al. [19] discussed carbon fiber tetrahedral truss cores by the hot press molding method and analyzed its properties on compressive and shear tests using experimental and finite element method (FEM) techniques. It was observed that the experimental ultimate compressive stress and shear stress for the composite was 3 MPa and 0.4 MPa, respectively, and these values coincide with FEM. Rashed et al. [20] reviewed the various available methods for the fabrication of a lattice core and its mechanical properties. Dong et al. [21] adopted the vacuum brazing technique to fabricate an octet lattice core using Ti–6Al–4V alloy sheets with a variation of relative density of 2–16% and analyzed its properties on compressive and shear strength. The highest ultimate compressive strength and shear strength were found in 16% of the relative density structures. Ullah et al. [22] investigated the compressive and shear properties of the Kagome truss core fabricated with Ti-6Al-4V using a selective laser melting method. The study was performed on two types of Kagome core, i.e., 1.2 mm truss diameter (angle = 55°) and 0.5 mm diameter (angle = 60°). It was observed that the ultimate compressive strength of the 1.2 mm truss diameter is 7.4 times stiffer than the other type of Kagome core. George et al. [23] investigated the effects of variation of relative density of polymer pyramidal truss cores with carbon fiber reinforced on the mechanical response. The core structures were fabricated using the snap-fitting method with a range of 1–10% of relative density. It was recorded that when the relative strength increased from 1 to 10, there was a maximum of 7.5-fold increase in shear strength.

#### 2.1.2. Foam Core

Foam cores are mostly used in critical engineering applications, for instance, in aircraft, automobile, building, nautical, and spacecraft applications due to their lightweight and better crashworthiness capability. These are open, closed, or no-cells types and the most used foam cores are polyvinylchloride (PVC) [24], polymethacrylimide (PMI) [25], and polyurethane (PU) [26]. In addition to this open and closed cell metal foam, aluminum foam can also be employed to achieve higher stiffness and fire resistance properties in conjunction with lightweight properties. Figure 4b shows the typical foam core [27]. The following is the brief literature survey in the context of foam core.

Long et al. [28] reported on the polyurethane foam core with densities of 52 and 75 kg/m^3^, tested using a drop weight impact instrument at different impact energies of 7, 20, 25, 35, and 45 J. It was observed that sandwich cores made with hardcore were more susceptible to delamination compared with softcore. Kazemi et al. [29] evaluated energy absorption characteristics of graded PU foam core under quasi-static punch load. CoDyre et al. [30] investigated the influence of the polyisocyanurate (PIR) foam core’s density (32, 64, and 96 kg/m^3^) on the axial compression properties of sandwich panels and found an increase in peak load by varying the core density.

#### 2.1.3. Corrugated Core

**Figure 5:**
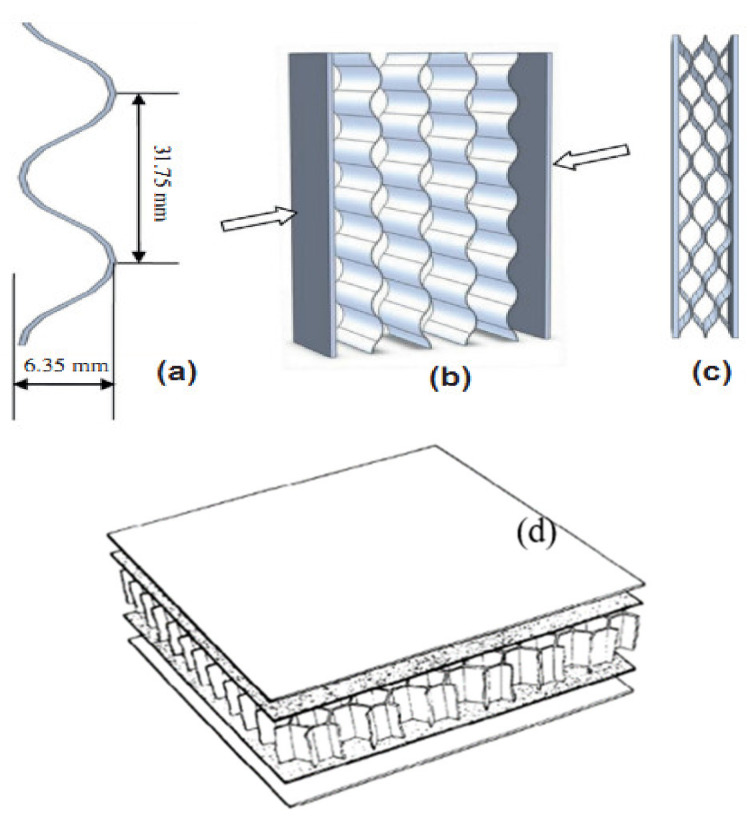
(**a**) Corrugated core sheet dimension; (**b**) Assembly procedure [31]; (**c**) Final sandwich panel; (**d**) Honeycomb sandwich structure [44].

These cores are unidirectional support open to one side. The cardboard corrugated cores are widely used in the packaging industry due to their shock absorbing capabilities and low cost. Figure 5a–c shows the typical corrugated core [31]. The following is the brief literature survey in the context of the corrugated core.

Kavermann et al. [32] investigated the mechanical properties of the corrugated plywood core subjected to a compression test. The test was performed on a single- and double-layer corrugated core and found that a single-layer corrugated core has 34% additional compressive strength than a double layer. Xu et al. [33] investigated the mechanical response of three-dimensional corrugated cores embedded with carbon fiber/epoxy face sheets, which were fabricated by an auto-cutting technique. It was observed that the graded parameter greatly influences the compressive strength. The small-graded size of 0.17 mm showed the highest compressive strength of 2.37 MPa, which decreased by 57% for 0.50 mm graded core due to buckling.

Magnucka et al. [34] studied the mechanical and vibration properties of the trapezoidal corrugated core using the analytical method. It was observed that the stiffness of the plate is inversely related to the length of the corrugation and the difference in natural frequency is in the range of 0.4–5.8% when the length of the corrugation increases from 1104 to 2392 mm. He et al. [35] reported the low-velocity impact performance of the aluminum alloy corrugated core with a variation of thickness and thereof relative density, from 5.52 to 8.83%. It was observed that peak values of impact load vs. time curve ascend with an increase in energy and relative density. Yang et al. [36] studied the modal analysis of axial- and circular-shape corrugated cores made with carbon fiber reinforced weave fabrics using the hot press molding technique. It was observed that the circular corrugated cores have exhibited the highest natural frequency, which was increased 3-fold at the 10th mode.

#### 2.1.4. Honeycomb Core

These are cellular structures with bidirectional support that may be open to thickness direction or length direction. The structures when open to thickness direction provide bending resistance, and cushioning ability when opened in length direction. This makes it possible for the honeycomb to achieve high anisotropy in a different direction. The unit cell in the honeycomb core can be triangular, square, or hexagonal [37]. The hexagonal cell can also be further divided into a regular hexagon, a reinforced hexagon, over-expanded or under-expanded [38]. The materials used for the fabrication of the honeycomb core were aluminum [39], thermoplastic polyurethane [40], Nomex [41], carbon fiber [42], foam [43], etc. Figure 5d shows the typical honeycomb core [44]. The following is the brief literature survey in the context of the honeycomb core. Zhang et al. [45] reported the dynamic impact behavior of the aluminum honeycomb core packed with expanded polypropylene foam with varying foam densities (20, 40, and 60 kg/m^3^) and impact velocities (2, 2.6, and 3.2 m/s). It was observed that with the variation of foam density, there is a decrease in the energy absorption capability of about 3% for 60 kg/m^3^ compared to the bare core.

It was also recorded that with an increase in velocity, there is an increase in energy absorption ability. Chen et al. [46] investigated the in-plane energy absorption capability of 3D printed hierarchical honeycombs made with Vero White polymer filament using a uniaxial compression test. The study was performed with varying relative densities, i.e., 0.16, 0.32, and 0.55. It was observed that the ultimate specific compressive load for 0.16 is 12 kN-m/kg, which decreased to 25 and 66% for the 0.32 and 0.55 relative densities, respectively. The decrease in ultimate strength with an increase in relative density is attributed to the decrease in stiffness, which results in buckling and cell wall fracture. Wang et al. [47] studied the inclined honeycomb core, with the inclination angle ranging from 0 to 90° and made with aluminum foil. It was observed that the inclination angle influences the plane stress vs. the compression ratio, and the plane stress decreases significantly after 45°. Sun et al. [48] adopted an interlocking method to fabricate three types of aluminum honeycomb cores, i.e., normal, grid, and a combination of both, and performed an in-plane compression test.

It was seen that the highest specific stiffness is observed for the third type of honeycomb core, which decreased by 55 and 70%, compared to the grid and normal honeycomb core, respectively. The rationale was explained in terms of interfacial toughness and high moment of inertia with the addition of a thick grid.

### 2.2. Innovative Core Structure

The innovative cores can be broadly categorized into derivate, hybrid, hollow, hierarchical, graded, folded, and smart core. The subsequent section discusses the innovative cores in detail.

#### 2.2.1. Derivate Core

The derivate core is further classified into auxetic, Y-shaped, and egg-box cores, which are discussed below.

##### Auxetic Structure

**Figure 6:**
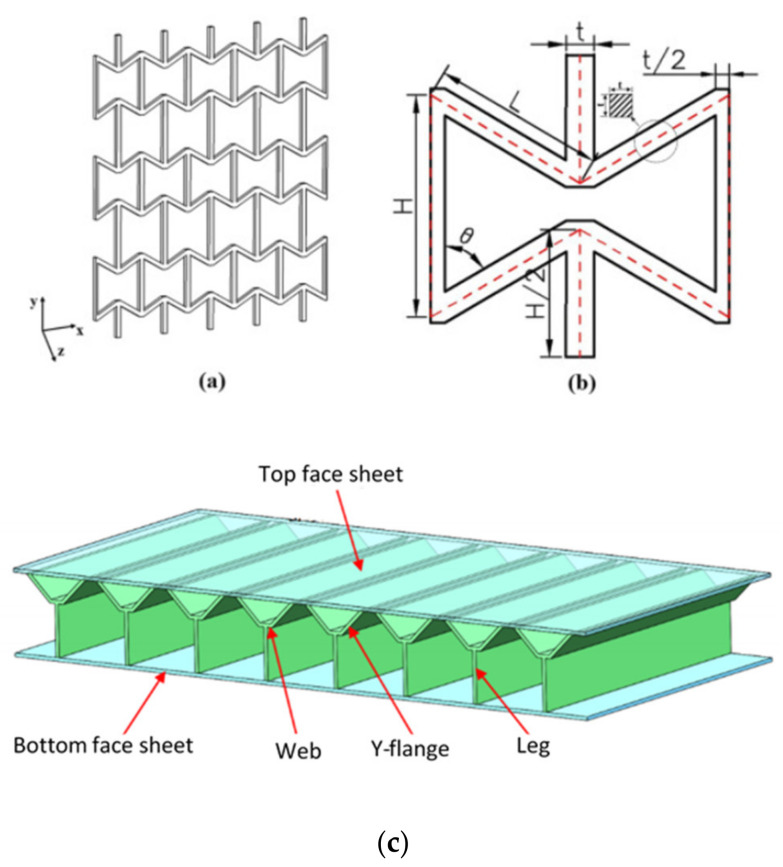
(**a**) 2D representation of auxetic cellular structure; (**b**) Representative unit cell [49]; (**c**) Y-shaped core [52].

Auxetic are the structural meta-materials that contradict the general theory that the structure swells under compression rather following shrinkage behavior. Alternatively, it shows an adverse Poisson’s ratio effect. The typical core with an auxetic structure is shown in Figure 6a and its unit cell is shown in Figure 6b [49]. Wang et al. [49] adopted the strain-based homogenization method to perform elastic analysis of a re-entrant-based auxetic structure. It was revealed that aspect ratio, length ratio, and re-entrant angle are the three geometric parameters that influence the elastic properties. Amaya-Amaya et al. [50] carried out acoustic properties measurements of re-entrant auxetic structures with polylactic acid (PLA) reinforced with keratin fiber materials fabricated via a 3D printing route. It was observed that the existence of keratin materials in the free space of the PLA/keratin composite significantly improved the sound absorption coefficient. The study on the influence of cell numbers on the effective elastic properties of the auxetic structure was carried out by Carneiro et al. [51] using FEA analysis via ANSYS 17. It was noted that the addition of re-entrant cells in the composite causes an exponential rise in Poisson’s ratio and a reduction in the normalized Young’s modulus.

##### Y-Shaped Core

Figure 6c shows the typical Y-shaped core sandwich structure [52]. The cores were manufactured through the hot-press molding method and accessed its performance through the edgewise compression method for different relative density samples. It was observed that there was an enhancement of 587% in failure load when the relative density was tuned from 5.3 to 10.5%. The dominant failure mode observed was macro buckling. Yan et al. [53] reported on the energy absorption characteristics of foam-filled metallic Y-shaped core sandwich panels under compressive strength. It was noted that the specific energy absorption of the foam-filled structure was increased 20-fold compared to that of the empty panel. Yiru et al. [54] investigated the compressive behavior of the Y-shaped core and compared the results with three different-shaped cores, i.e., the X, A, and W-Shaped cores. The total energy absorption and energy absorption efficiency followed the trend: EA(A) > EA(X) > EA(Y) > EA(W) and EAE(A) > EAE(Y) > EAE(X) > EAE(W). The thickness effect was also analyzed and it was observed that the thickness had a greater influence on the sandwich panel.

##### Egg-Box Core

**Figure 7:**
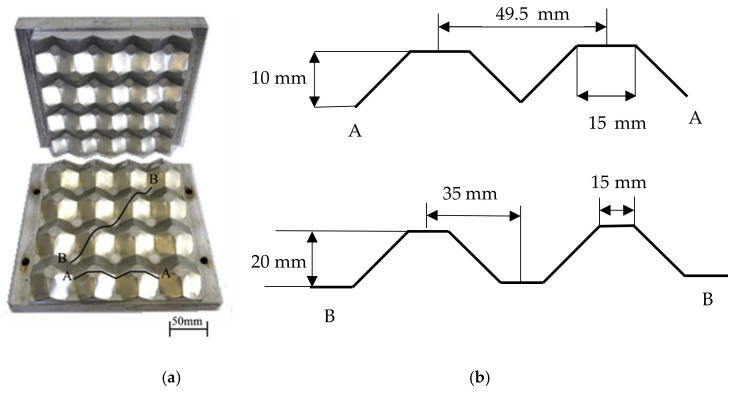

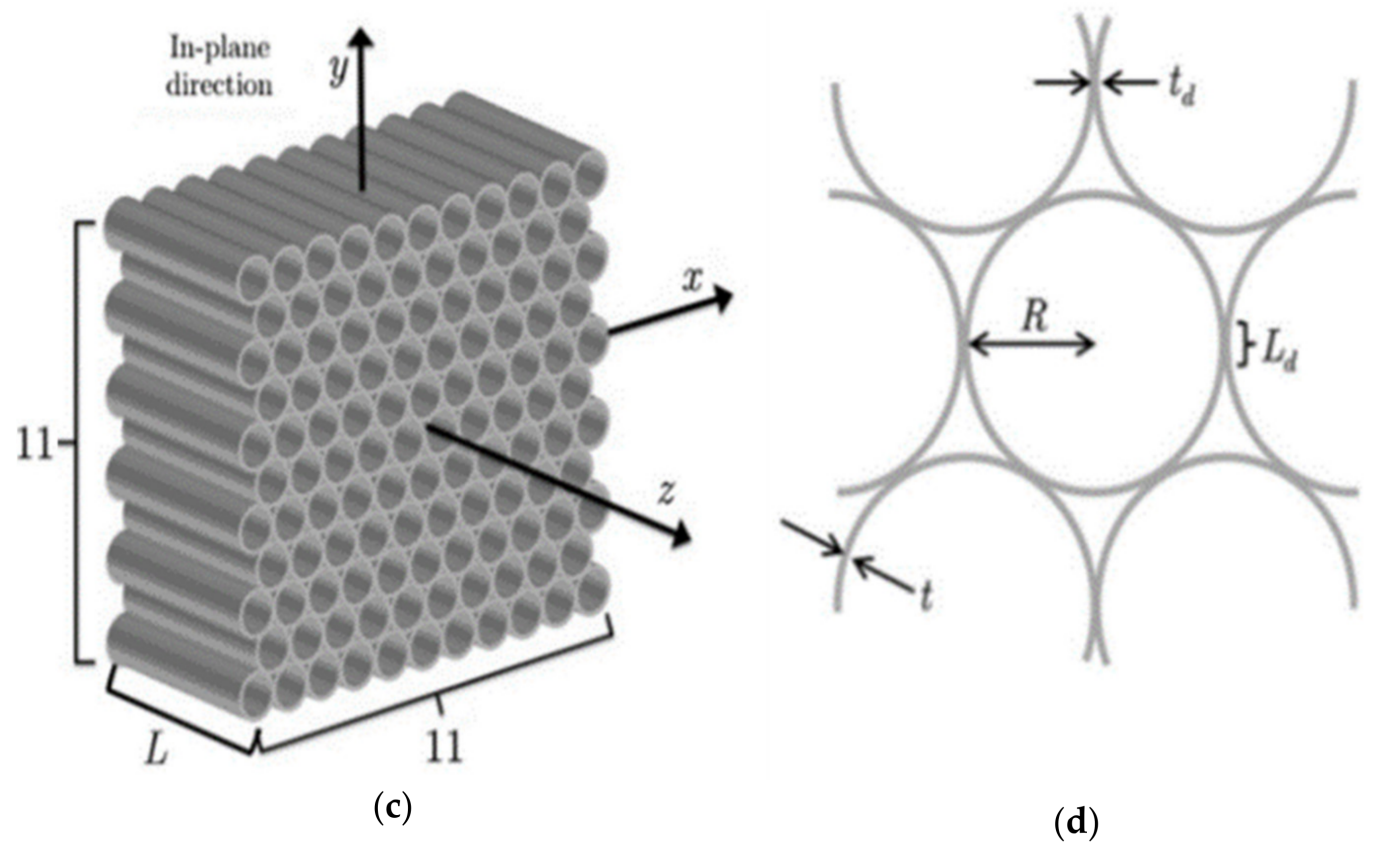
(**a**) Photograph of mold used to fabricate the GFRP core; (**b**) Profile of the cross-section of a GFRP core; [55] (**c**) Sketch of a 11 × 11 size circular cell honeycomb; (**d**) Details of the microsection with relevant dimensions, R: cell radius, t: wall thickness, L: cell length, td: double-wall thickness, L_d_: bond length (right) [58].

The egg-box core is a nature-inspired engineered structure that is developed with a three-dimensional dimpled shell shape. The application area of such structures includes the automotive, aeronautical, naval, high-speed train, sports equipment, and architecture industries. The reason for the implementation of the egg-box core in the above critical areas is owed to its high-energy absorption, superior vibration absorption capability, and outstanding heat dissipation efficiency. Figure 7a,b shows the typical egg-box core [55]. Haldar et al. [55] carried out mechanical behavior egg-box sandwich structures fabricated via the hot press molding method. The material for the manufacturing of the core was glass fiber reinforced epoxy (GFRP) with carbon fiber reinforced epoxy (CFRP) prepreg materials. The quasi-static and dynamic test revealed that specific energy absorption (SEA) was improved swiftly with the increase in cell wall thickness. It was also noted that the dynamic SEA values were superior to the quasi-static ones due to the rate sensitivity of the material. Cai et al. [56] reported on the plastic forming analysis of the egg-box core structure via experimental and finite element simulation approaches. It was observed that the failure during the formation of the egg-box was primarily due to fracture. Fathers et al. [57] investigated the out-of-plane quasi-static crushing properties of the egg-box structure with two different geometries, such as cube and diamond strip core. It was noted that the diamond strip had 24% and 41% superior peak and average stresses when compared to a simple cube-type egg box core.

#### 2.2.2. Hollow Core

##### Circular Honeycomb

Circular tube honeycombs are suitable for blast resistance and as protective structures due to their excellent energy absorption ability and well-regulated deformation pattern. Figure 7c,d shows the typical circular honeycomb core [58]. Liu et al. [59] investigated blast resistance and parametric analysis of sandwich plate honeycomb filled with circular tubes (SP-HFCT). The maximum back face sheet deformation was seen for SP-HFCT when compared to the general honeycomb plate (GHP). Cernescu et al. [60] reported on the mechanical properties of the honeycomb core with a circular cell geometry. The half-cell was manufactured through plastic deformation, and the two halves were subsequently joined through laser welding.

The compression and shear stiffness were analyzed by taking a unit cell, which varies when subjected to the loading direction, signifying an orthotropic behavior possessed by honeycomb structures with circular cells. Yang et al. [61] tested the dynamic crushing properties of the novel circular cell honeycomb, i.e., the petal-shaped honeycomb (PSH) structure, in the in-plane direction. It was observed that at 1 and 35 m/s of impact velocities, the specific energy absorption (SEA) was increased by 71.3 and 80.4%, respectively, when compared to the circular cell honeycomb structure.

##### Corrugated Core

**Figure 8:**
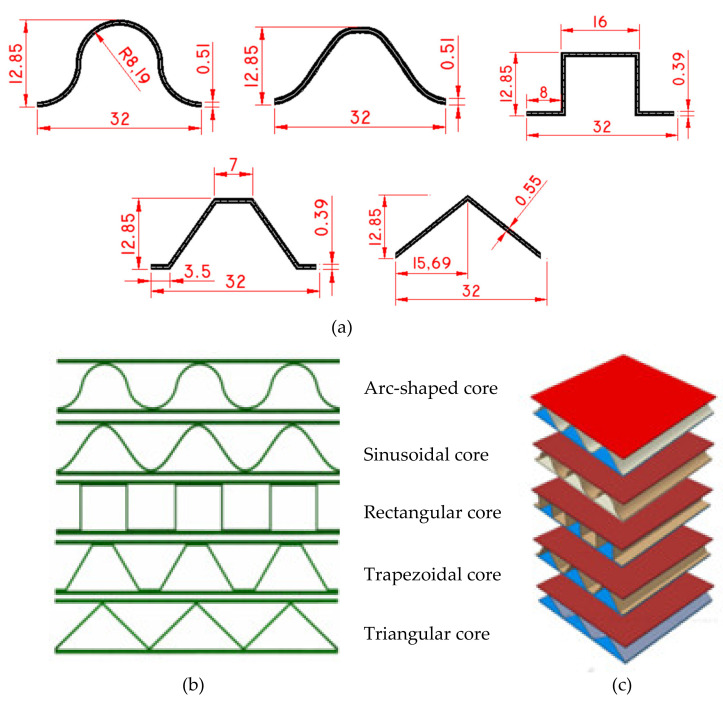
(**a**) Sketch of five types of unit cell; Maps of the five sandwich panels with corrugated-core geometric configuration: (**b**) Cross-sections and (**c**) Axonometric drawing [62].

The corrugated core structures have attracted much attention in recent years because they can greatly enhance the energy absorption capability of such structures when the proper corrugated parameters are selected. Figure 8a–c shows the typical corrugated core [62]. Li et al. [63] investigated the fabrication and performance of corrugated-core-based sandwich cylinders (CSCs) and lattice truss core sandwich cylinders (LTSCs). The compression test was conducted, and it was noted that there is an enhancement in the performance of CSCs by 50% when compared to LTSCs. Ma et al. [64] reported on the crashworthiness performance of the corrugated core tubular structure via two types of inner rib designs (i.e., X and Y-shaped) using the finite-element based LS-DYNA software. It was seen that the crushing force efficiency (CFE), the specific energy absorption (SEA), and the undulation of load-carrying capacity (ULC) values for Y-shaped tubes are superior compared to X-shaped tubes. Rejab et al. [65] tested the mechanical performance of the corrugated-core sandwich beam through triangular profile unit cell. The results suggested that the overall and local collapse behavior was influenced by the number of unit cell arrangements and cell wall thickness.

#### 2.2.3. Hybrid Core

**Figure 9:**
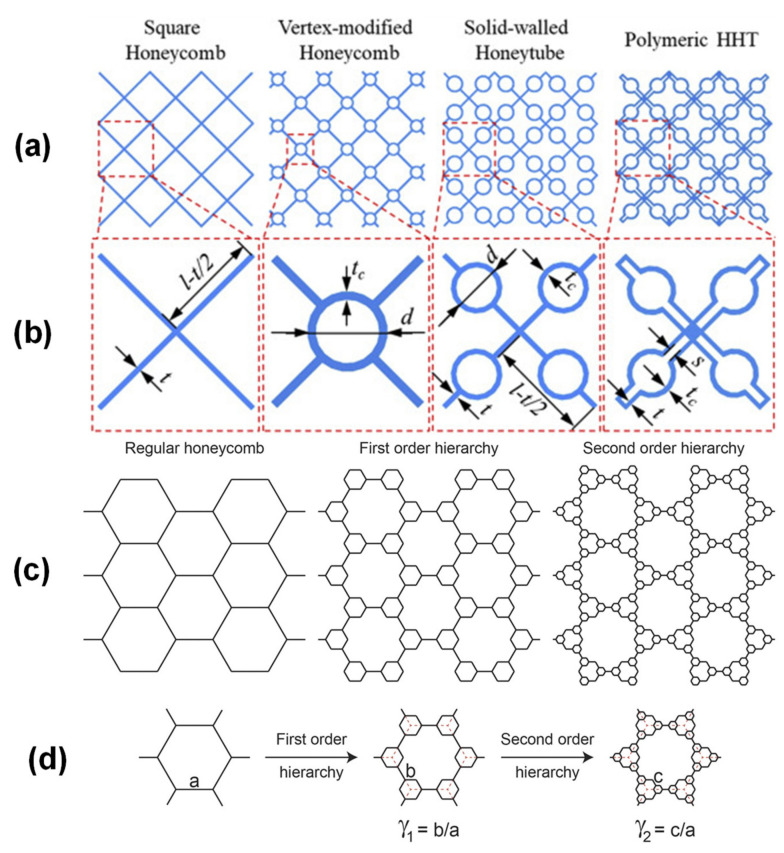
(**a**,**b**) Illustration of HHTs together with square honeycomb, vertex modified honeycomb, solid walled honeytubes, and polymeric HHT [66]; (**c**) Regular, first order, and second order hierarchy; (**d**) Process of converting unit cell from first order to second order hierarchy [70].

Recently, through inserting various materials into the interstices of monolithic cores, the so-called hybrid core can satisfy the additional functionality requirement of severe engineering applications, such as ballistic and blast resistance, impact noise, and vibration absorption, etc. Figure 9a,b shows the typical hybrid core [66]. Yungwirth et al. [67] reported the ballistic performance of the monolithic truss core filled with polyurethane, alumina, and aramid fiber. It was observed that the addition of alumina enormously enhanced the penetration resistance of the monolithic truss core. Yan et al. [68] introduced a hybrid sandwich structure made with a metallic-corrugated core filled with aluminum foam. The performance was analyzed experimentally through the transverse direction using a three-point bending test. It was found that the filling of aluminum foam into the monolithic plate led to an increase in the bending strength and stiffness of the sandwich plate to great extent. Han et al. [69] investigated the interstices of aluminum corrugations through a meticulousness-cut trapezoidal aluminum honeycomb plate. It was observed that the compressive, shear strengths along with the energy absorption of the sandwich plate were greatly enhanced when compared to an empty honeycomb-corrugation core.

#### 2.2.4. Hierarchical Core

The concept of structural hierarchy in regular geometry is called the hierarchical core. It is theoretically established by replacing the cell walls of typical honeycombs through lattices of Kagome, and thanks to a triangular structure the stiffness is enhanced by about two orders of magnitude. Figure 9c,d shows the typical hierarchical core [70]. Chen et al. [71] carried out a numerical approach for demonstrating structural hierarchy in typical honeycomb results to augment different properties such as heat resistance, thermal anisotropy, along with mechanical performance. The results exposed that the combined thermal mitigation and load-carrying capability of the hierarchical honeycomb designs are endorsed to the introduction of structural hierarchy. Sun et al. [72] investigated the dynamic behavior of sandwich beams with cores of hierarchical honeycomb design under blast loading. It was observed that the maximum deflection at the back face sheet of the hierarchical honeycomb was smaller than that of a typical honeycomb at a higher level of blast load. Chen et al. [73] proposed parametric analyses that influence crucial parameters on the local buckling such as stress and strength-to-density ratio. The results also suggested that the energy-absorption properties are enhanced with an increasing number of hierarchies.

#### 2.2.5. Graded Core

**Figure 10:**
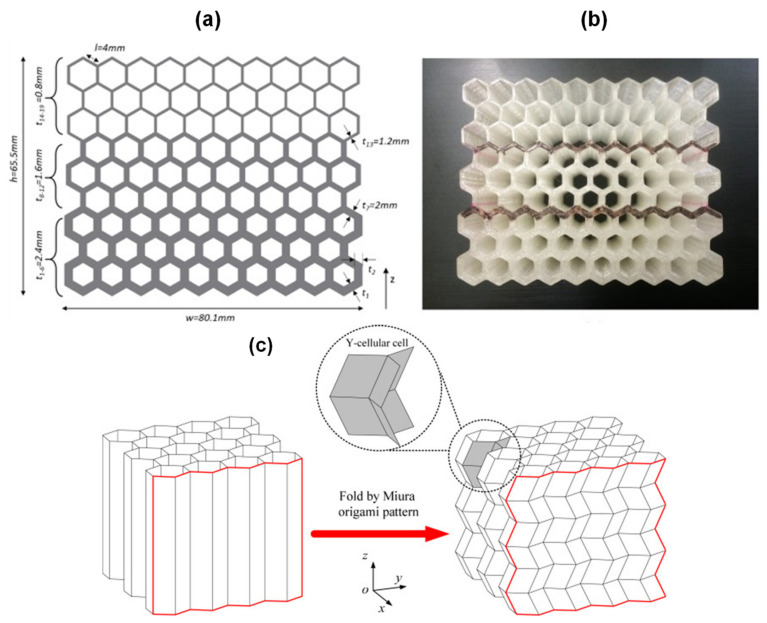
(**a**) Design details of 3 stage graded hexagon; (**b**) The test specimens produced by FFF 3D printing process [40]; (**c**) Plan used in the construction of pre-folded honeycomb [78].

With the growing necessity of lightweight and crash-protecting structures, an innovative class of structural configuration, specifically functionally graded structures (FGSs) whose density changes continuously in one direction, has recently attracted attention due to the specific advantage of tailorable energy absorption and blast protective ability [74]. Figure 10a,b shows the typical graded core [40]. Bates et al. [40] explored four types of density-graded honeycomb structures, such as two, three, and five stages, and the continuously graded honeycomb structure, which is fabricated via thermoplastic polyurethanes filament using 3D printing technology. The energy absorption capability was tested through quasi-static and cyclic compression tests. It was perceived that the energy absorption range was wide for all the graded structures when compared to uniform structures owing to higher strain-to-densification and the non-linear correlation with that of density and energy absorption. Zhu et al. [75] introduced a double functionally graded tube (DFGT) structure by filling the functionally graded honeycomb (FGH) in a tube of the functionally graded thickness (FGT). It was noticed that the DFGT design enhanced the global bending resistance when subjected to oblique loading, which generates a wider progressive region for superior energy absorption properties. Yu et al. [76] reported the structural performance of in-plane gradient honeycomb sandwich plates subjected to quasi-static and dynamic loading. It was observed that when the in-plane gradient increases (positive gradient), the strength, stiffness, and plastic energy dissipation of the sandwich plate are improved significantly. Sahu et al. [77] investigated in-plane static and dynamic compressive behavior of three types of novel gradient structures, i.e., thickness, length, and hybrid gradient structures. Among all the samples, the hybrid gradient structure has a superior damping ability owing to a lower cells-per-honeycomb surface area (C/HSA).

#### 2.2.6. Folded Core

The core structure with a periodic bent pattern is well-known as folder core. The folded cores not only resolve the problem of humidity accumulation owing to their exposed ventilation channels, but also serve as efficient energy absorption structures. Figure 10c shows the typical folded core [78]. Heimbs et al. [79] analyzed the mechanical properties of carbon fiber reinforced plastic sandwich structures using a folded core subjected to low velocity impact experimentally, which was numerically validated. The test results exhibited localized failure when exposed to impact loads, due to global bending deformation of the top face layer. Kintscher et al. [80] analyzed the stiffness and failure behavior of the folded core under combined compressive and transverse shear load. The cores were fabricated using Nomex paper, which was coated with an epoxy resin. It was observed that the compression stiffness reduces when the initial shear deformation was increased. Lebee et al. [81] proposed a new plate theory using a homogenization scheme via the bending-gradient plate theory, which was extended to classical periodic plate theory. It was observed that the skin distortion was greatly influenced by the shear force.

#### 2.2.7. Smart Core

Smart core materials efficiently utilize the design advantage of the sandwich structure into lightweight load-bearing smart composite sandwich structures for a wide range of applications, which includes noise and vibration control to mechanical power transmission and structural health monitoring systems. The smart core sandwich structure can be classified according to the type of material used, such as: piezoelectric, shape memory polymer (SMP), magnetorheological fluid (MRF), magneto-rheological elastomers (MREs), electrorheological fluid (ERF), and electrorheological elastomer (ERE) sandwich beam.

##### Piezoelectric

**Figure 11:**
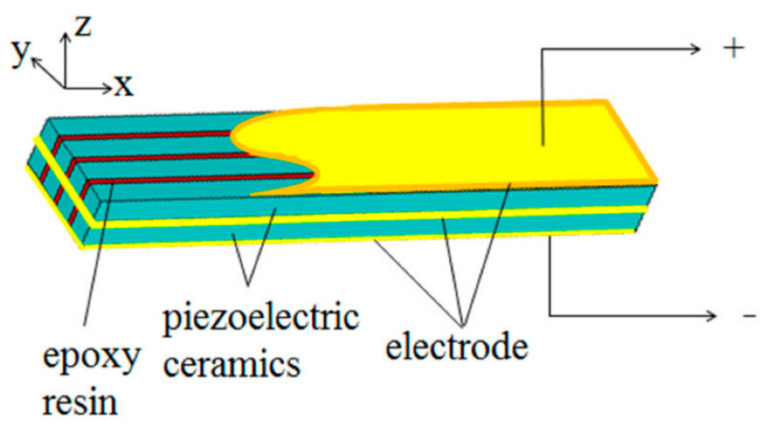
Structure of the piezoelectric composite bi-laminated vibrator [82].

These sandwich beams are fabricated with a lightweight core integrated with two layers of stiff face sheets accompanied by patches of piezoelectric sensors on the face sheets as shown in Figure 11 [82]. The three-layer sandwich beam discussed above is then clamped/bonded together to achieve a piezoelectric sandwich beam. Moradi-Dastjerdi et al. [83] investigated the free vibration analysis of a multifunctional smart sandwich plate (MSSP) with layers of active piezo-ceramic skin integrated with a passive lightweight core reinforced with CNTs. The results show that with the addition of CNTs (up to 0.5%), the natural frequency of MSSPs was significantly improved. Li et al. [84] adopted a numerical method to investigate the active vibration control of the pyramidal lattice core with a patch of piezoelectric material on the top and bottom layers of the face sheet. The velocity feedback control (VFC) and the linear quadratic regulator control (LQRC) methods were adopted for the numerical analysis, and it was observed that, for both methods the required maximum control voltage matches with the first mode; however, for other modes, it is much larger. Beheshti-Aval et al. [85] proposed FEM for piezoelectric beam sandwich structures with different widths via the high-order global–local theory method. It was seen that by adopting this method, the unknown parameters are reduced in addition, independently of the number of layers in the sandwich construction.

##### Shape Memory Polymers (SMPs) and Shape Memory Alloy (SMA)

**Figure 12:**
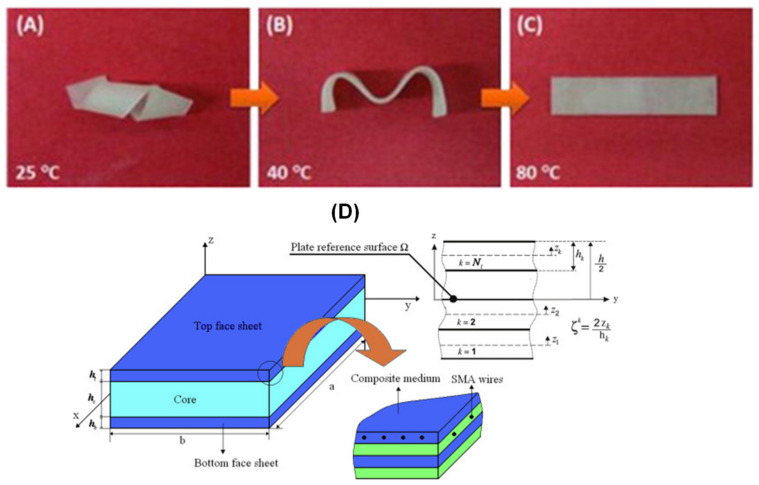
The sequential recovery of the epoxy/polycaprolactone composite (**A**) Deformation from a temporary shape, (**B**) Deformation to temporary shape, (**C**) Deformation to a permanent shape [86], (**D**) Geometry and coordinate systems of sandwich plate with SMA hybrid composite faces [87].

Shape memory polymers (SMPs) and shape memory alloy (SMA) are the unique class of materials that can recover their shape when an external stimulus is applied. Figure 12a–e shows the typical SMPs and SMA core [86,87]. Butaud et al. [88] investigated the damping performance of tert-Butyl Acrylate (tBA)/5 wt.% of the poly-ethylene glycol dimethacrylate (PEGDMA) SMP core with an aluminum face sheet. The result suggests that the controlled heating rate of the SMP core enables the damping of the structure for a wide-ranging frequency. John et al. [89] proposed a sandwich with an orthogrid stiffened SMP-based syntactic foam core and tested the impact damage analysis. There were two levels of pre-strain specimens, i.e., 3 and 20% were used. It was noticed that the maximum impact load for the 20% pre-strained specimen is about 17% higher than its 3% pre-strain specimens. This implies that the growth in the pre-strain level results in densifying the foam and hence stiffening the samples, leading to an increase in the load-sustaining ability. The improvement in stiffness and shape recovery behavior of two types of SMP-based sandwich structures, such as aluminum/SM-polyurethane/aluminum and steel/SM-polyurethane/steel were carried out by Li et al. [90]. It was observed that shape recovery stress was improved significantly for SMP-based sandwich structures.

##### Magnetorheological Fluid Sandwich Beam

**Figure 13:**
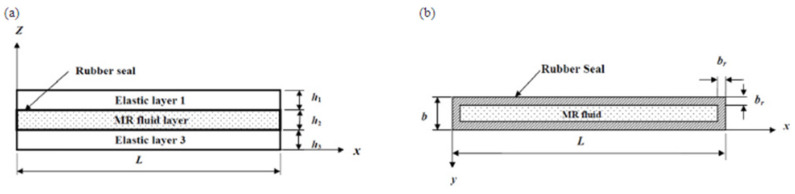
(**a**) MR fluid sandwich beam; (**b**) Plane view of MR fluid layer [91].

Magnetorheological fluid (MRF) is a group of smart materials whose viscosity or rheological trait changes swiftly and can be controlled in the presence of applied magnetic field. An MRF sandwich beam is made by placing two layers of continuous elastic structure with the MR fluid core shown in Figure 13a,b [91]. MRF has a novel tendency to change state conversely from solid to liquid depending on the magnetic field which is attained by the shift of iron particles. The MRF core exhibits significantly higher dynamic yield strength and greater insensitivity to temperature variations compared to ER fluids. Sternberg A et al. [92] studied the design and tested the MR damper using the FEM tool and found that the developed solution using FEM provides a satisfactory experimental outcome. Kim. S et al. [93] reported that MRF can be successfully engaged in robotic surgery because it can generate repulsive force during tissue surgery. Kaluvan S. et al. [94] proposed an MRF, placed between a pair of electrode coils, which can be applied to the motion control of an actuator by working on the principle of magnetic extension as well as contraction by MRF.

##### Magnetorheological Elastomers (MREs) Core

**Figure 14:**
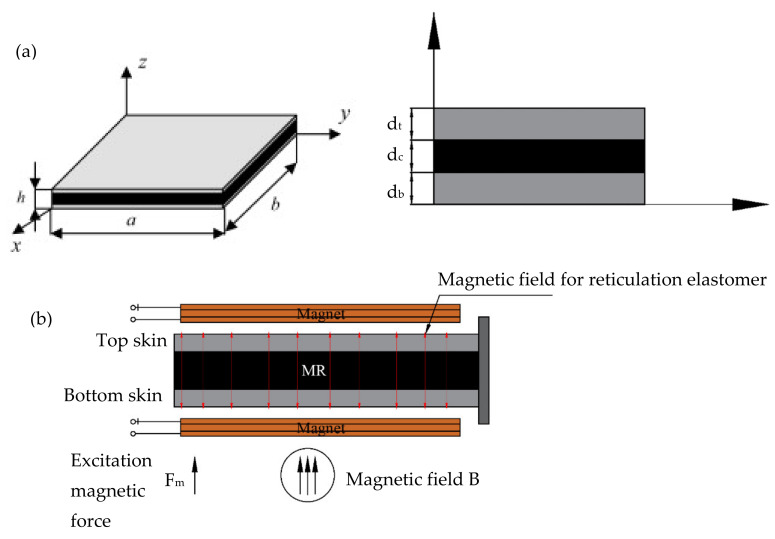
(**a**) Sandwich plate with the elastomer part; (**b**) Vibrating sandwich plate subjected to a perpendicular magnetic field [96].

Magnetorheological elastomers (MREs) are a class of smart material with electromagnetic composites made by magnetic particles embedded in an elastomeric matrix with a novel capability to change its mechanical properties, such as stiffness and vibration characteristics when it is being subjected to a magnetic field, and which reversibly modifies when the magnetic field is removed [95]. MREs are intended to provide high flexibility, be easily moldable, offer excellent durability, exhibit hyperelastic performance, and be able to provide desired mechanical and thermal properties. MREs have several applications, from highly developed synthetic muscles to vibration absorbers and modern sensors. Figure 14a,b shows the typical MREs sandwich beam [96]. Ni et al. [97] reported the micro-vibration control of equipment under speculative support motion. MREs fused in the sandwich pillar as the core have an impressive small-scale vibration concealment ability for diverse small-scale support motion excitations. Han, Y. et al. [98] studied the on-field stiffening result of MREs and found that MREs with additional iron particles generally performs better than the MR effect. Schubert, G. et al. [99] focused on the permeability study of MREs using the inverse modeling method and found that samples with a larger content of iron particles have superior permeability. Bocian, M. et al. [100] reported on the magneto mechanical properties of MREs and found that the increase in excitation force frequency indicates there is a change in stiffness. Kumar, T. P. et al. [101] investigated the dynamic study of the MRE-implanted sandwich plate using the finite element method and the Lagrange principle and the result showed that with the magnetic field, the natural frequency and modal loss factor of MREs increases.

##### Electrorheological Fluid (ERF) Core

**Figure 15:**
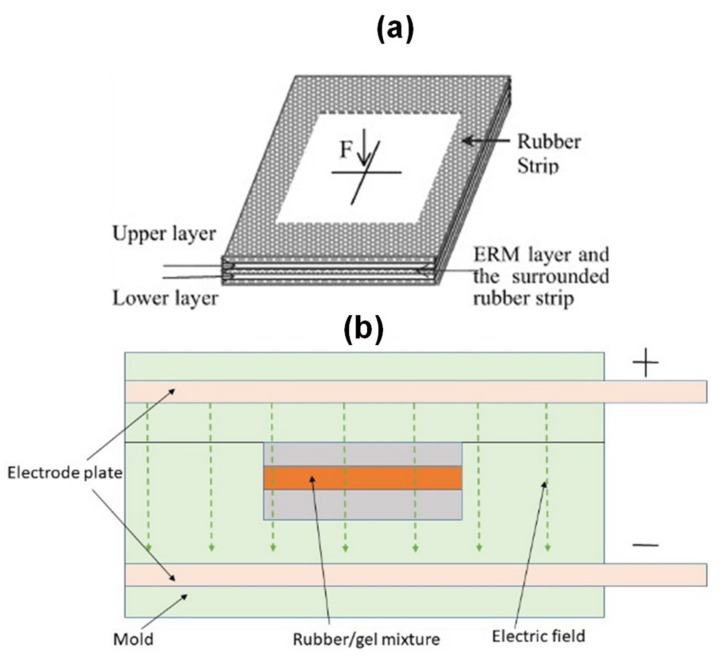
(**a**) Electrorheological fluid (ERF) sandwich beam [103]; (**b**) Electrorheological elastomer (ERE) sandwich beam.

Electrorheological (ER) fluids or viscoelastic layers are found among smart materials, with controllable rheological properties which exhibit noticeable reversible changes in their viscosity under the influence of an applied electric field which makes them suitable in adaptive dampers and intelligent structures, as well as in feedback control systems for robotics and automotive applications [102]. In general, the ERF sandwich beam is made by placing the ERF between two FGM layers. Figure 15a shows the typical ERF sandwich beam [103]. Lee, C.Y. et al. [104] studied the dynamic behavior of electrorheological material with electric fields between grooved surfaces and electrodes and found that the ER effect was improved when impressing denser rolled grooves on the surface of the electrode. Allahverdizadeh. A et al. [105] studied the dynamic behavior of the functional graded electrorheological fluid sandwich beam and found that at a constant electric field amplitude, the crest diminished with an increase in the functional graded material volume fraction index. Abu-Jdayil et al. [106] reported on ER fluid and its effect on rotational and slit flow, and showed that by implementing trevira mesh, the electrorheological effect can be improved. Vivas-Lopez [107] proposed a method for the modeling of the ER damper and found that the current model has 28.4% less error–signal ratio compared to the Eyring plastic model.

##### Electrorheological Elastomer (ERE) Sandwich Beam

The electrorheological elastomer is composed of natural rubber dispersed with polarizable particles. The ERE sandwich beam is subjected to change in the electric field. This change in electric field causes a change in the Young’s modulus of the structure. The ERE sandwich beam is best suited for transmission elements, shock absorbers, and engine mounts. Figure 15b shows the typical ERE sandwich beam. Gao. L et al. [108] studied starch/gelatin/glycerin-composite electrorheological elastomers and found that resistance and compression modulus individually could be enhanced with the weight fraction due to the fact that, under the electric field, starch particles form the chain structure of the matrix. Wang. B et al. [109] studied the synthesis and characterization of clay/gelatin ERE and found that clay particles demonstrate a preferential orientation inside the matrix, exhibiting anisotropy.

## 3. Composite Skin Material

**Figure 16:**
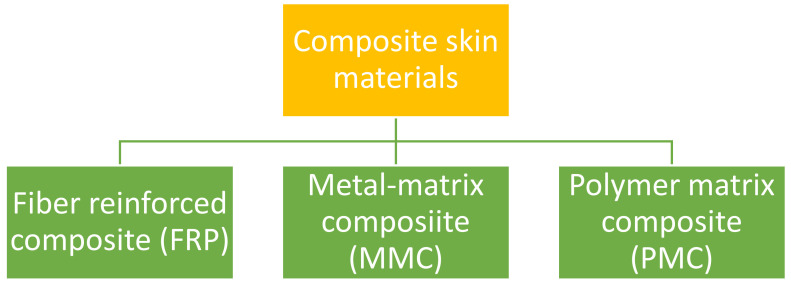
Classification of composite skin material.

The indispensable property of skin used in the sandwich structure is to resist in-plane shearing and out-of-plane compressive load and to prevent itself from bending and fracturing. Nearly all structural materials which are accessible in the form of thin sheets may be used to form the faces of the sandwich panel. The material for the face sheet should have good toughness, hardness, and impact resistance ability. The composite skin in this particular case can be well suited compared to virgin material that satisfies the above requirement [110]. The composite skin has recently shown applications in various industries. For instance, the panels in aircraft structures make use of composite steel, aluminum, or other metals, even though reinforced plastics are very often adopted in remarkable applications to reduce weight. The skin material is broadly classified into fiber reinforced composites, metal-matrix composites, and polymer matrix composites as shown in Figure 16, which is discussed in the following section.

### 3.1. Fiber Reinforced Composites

Fiber reinforced composites (FRCs) are a group of structural composites that consist of a reinforcing material, usually fibrous or particulate. Reinforcing materials, such as glass fiber, carbon fiber, and Kevlar fiber are available in the form of fibrous or particulate form. The matrix can be a thermoset or thermoplastic polymer. In FRCs, elevated strength and rigidity make them able to abolish the fiber direction. Recently, FRCs were widely used in sports apparatus, infrastructure applications, and racing bicycles wherein carbon fiber is the reinforcing material and thermoset polymer is the matrix used. The following is the brief literature survey in the context of FRCs: Barile et al. [111] reported on the mechanical characterization of carbon fiber reinforced plastic under tensile compression test with and without stitching. It is observed that with innovative stitching and fiber arrangement, there is a 14.5 and 11% increase in ultimate tensile and Young’s modulus, respectively. Noushini et al. [112] investigated synthetic fiber reinforced geopolymer concrete (FRGPC) on its mechanical and flexural performance. It was observed that FRGPC containing polypropylene fibers exhibited an average of 1 to 7% reduction in compressive strength compared to the plain geo-polymer concrete. Canche et al. [113] reported the mechanical properties of aramid fiber reinforced polypropylene–aluminum metal laminated composites and compared the results to that of plain aluminum and aramid fiber polypropylene sheets. It was found that the strain to failure of the fiber metal laminates (FMLs) increases by 230% and 400% compared to those of the plain aluminum sheet. Turk et al. [114] reported the thermo-mechanical investigation of acrylonitrile–butadiene–styrene (ABS) and polyamide (PA12) using the fused deposition modeling (FDM) and selective laser sintering (SLS) methods and studied the property along in (X) and out-of-plane (Z) directions. It is observed that at 90 °C, the average tensile strength of ABS decreased significantly by about 56% and 69% in X and Z directions, respectively, compared to that at room temperature.

### 3.2. Metal Matrix Composites

Metal matrix composites (MMCs) are among the fastest growing composite material family due to their potential tailored ability and high-temperature sustainability. Metal matrix composites are extensively employed in the aerospace, nautical, and automobile industries due to their significantly expanded strength, stiffness, outstanding biocompatibility, and weight diminishment when contrasted with that of conventional materials. In MMCs, the reinforcing material is metal or nonmetal, such as short carbon fiber, which can be continuous or discontinuous in a matrix of metal such as aluminum [115], magnesium [116], or titanium [117] suspended in a matrix. The followings is the brief literature survey in the context of MMCs.

Pazhouhanfar et al. [118] reported on the mechanical and microstructural characterization of aluminum matrix composites (Al6061) reinforced with titanium diboride (TiB_2_) ceramic particles of 3, 6, and 9 wt.%. It was observed that the addition of 9% reinforcement significantly improved the tensile strength and hardness by 41 and 93%, respectively. Ghasali et al. [119] investigated the effect of 15% TiC reinforcement on the mechanical and microstructural evaluation of the aluminum metal matrix composite using the sintering technique and compared it to that of conventional and microwave methods. It was observed that during the bending test, samples prepared from the sintering method showed an increase in load peak point by 150 and 66% compared to that of conventional and microwave methods. Shirvanimoghaddam et al. [120] studied the physical and mechanical characterization of aluminum matrix composite reinforced with boron carbide nanoparticle (B_4_C) varied from 5 to 15 vol% processed using stir casting at two different temperatures, i.e., 800 and 1000 °C. It was found that at 800 °C, the tensile strength was shown by 15% of B_4_C, whereas at 1000 °C, the highest tensile strength was shown by 10% of B_4_C, which increased by 13 and 15%, respectively.

### 3.3. Polymer Matrix Composite

Polymers are commonly used in the manufacture of pipes, storage tanks, gears, bearing materials, automotive body parts, medical instruments, and other applications due to their corrosion resistance, light weight, and low cost. Although polymers exhibit superior properties, they still possess some critical loopholes, such as lack of stiffness, low rigidity, and poor wear resistance. To overcome these, polymer composites are developed in due course. The polymer matrix composites (PMCs) are a new class of composite with improved properties compared to those of parent polymer by the addition of fillers. In polymer composites, the reinforcing material may be made of fibers, flakes, platelets, spheres, or other forms in a matrix of polymer, such as high-density polyethylene [121,122], polypropylene [123], and ultra-high molecular weight polyethylene [124]. The fillers in the PMCs may be inorganic minerals, namely graphene nanoplatelets [125,126], multi-walled carbon nanotubes [127], boron nitride [128], nano-diamonds [129], aluminum oxide [130], calcium carbonate [131], or organic materials such as argan nutshell [132], almond shell [133], or sisal fiber [134]. The followings is the brief literature survey on the context of polymer matrix composites. Li et al. [135] investigated the mechanical properties of the polymer composite metal hybrid (PMH) with PA 66 and HC 340HSS steel using injection molding and spray technology. It was observed that the specific strength of PMH was improved by 39 and 65%, compared to that of pure metal. Fu et al. [136] reported on the mechanical properties of polypropylene polymer composite reinforced with Kevlar fiber and fabricated by the melt mixing process. It was found that the addition of 10 wt.% of Kevlar fiber resulted in the enhancement of tensile strength of PP by 57%. Badgayan et al. [137] reported on the tribological properties of HDPE reinforced with MWCNT and BNNP nanoparticles using the mechanical mixing and molding process. It was concluded that the 0.25MWCNT/0.15BNNP composite combination showed the best wear resistance capacity among those of composites and hybrids.

Table 1 shows the different skin material available for composite skin fabrication.

## 4. Joining Technique

The performance of load bearing, lightweight sandwich structures requires a novel joining technique to accomplish the prime requirement of firm amalgamation skin with the core.

**Figure 17:**
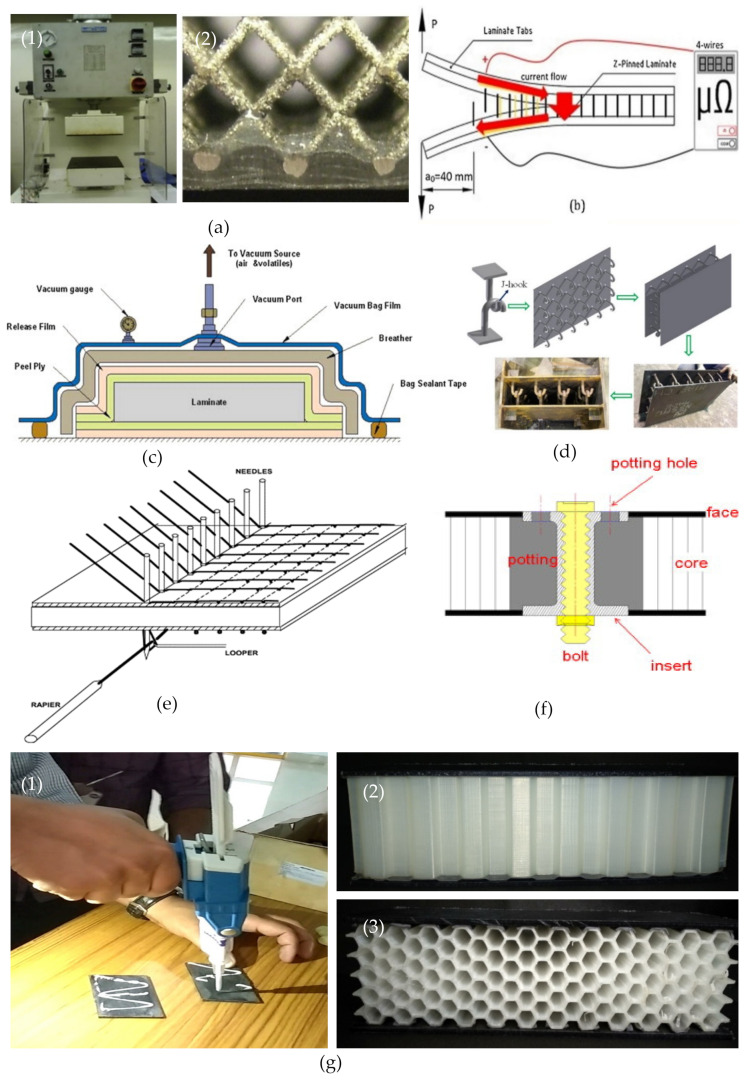
(**a**) Heated press (1) manufacture setup (2) skin–core bond details of SS316L lattice core and CFRP skins [158], (**b**) Z-pinning technique [159], (**c**) Vacuum beg setup [160], (**d**) J-hooking technique [161], (**e**) Stitching technique [162], (**f**) Bolting technique [163], (**g**) Adhesive technique; (1) Adhesive gun; (2) Sandwich construction in out-of-plane; and (3) In-plane direction [110].

The joining techniques play a vital role in the fabrication of a sandwich structure, which can act as a potential energy transforming unit from the skin to the core. The joining procedures adopted by the researchers are wide and diverse depending on their material properties, such as place of application and overall strength requirement. Among the joining technology, one can find heated press [158], vacuum bagging [159], Z-pinning [160], J-hooking [161], stitching [162], bolting [163], and adhesives [110] are usually adopted in the fabrication of sandwich structures, which is illustrated in Figure 17. The following is the brief literature survey in the context of joining techniques.

Feng et al. [164] investigated Kenaf/glass reinforced hybrid composites on the shearing failure strength test with bolted joint. The test was conducted in a heated chamber with a temperature sweep from 25 to 60 °C. It was observed that an increase in the preload moment of the bolted joint improves the load carrying capacity. Wei et al. [165] performed single lap joint testing on CFRP steel with two types of adhesives, i.e., 7779 and MA830. It was noted that the joint strength is mostly dependent on overlap length and type of adhesive, and the joint with the 7779 adhesive showed a 3 kN higher failure load than the MA830 adhesive. Chen et al. [166] adopted the resin transfer molding method to fabricate sandwich structures with PVC foam core and glass aramid fiber face sheet. The material used for resin was epoxy and curing agent at 10:3 ratios. It was observed that the sandwich panels made with chopped fiber toughening provided a strength about 0.06% higher than that of the virgin sample.

## 5. Testing and Performance

### 5.1. Compression Test

The compressive test for a sandwich core panel is performed according to ASTM C 365 standard [167]. Compressive strength and modulus are usually determined from the above test with a nominal size of the specimen as 75.6 × 75.6 mm. The compressive strength deals with the ultimate compressive stress that a sandwich structure is proficient in withstanding without undergoing fracture, whereas modulus is the slope in the stress vs. strain curve, which measures the stiffness of the structure. The compressive strength and modulus are measured using the following equations [168].

(1) $$\sigma_{c}=\frac{P_{c}}{A_{c}}$$

(2) $$E_{c}=\frac{m.t}{A_{c}}$$

The symbols used in the above equation may be referred to in the literature of Zaharia et al. [168].

The energy absorption measured the area under the stress–strain diagram [169]. Dikshit et al. [170] performed out-of-plane compressive strength analysis of the 3D printed composite. There were two types of structures considered, i.e., vertical pillar based corrugated sine wave (VPSC) and corrugated trapezoidal (VPTC). It was noted that the ultimate compressive strength (UCS) of the VPSC structure was improved by 16.6% when compared to the VPTC structures.

**Figure 18:**
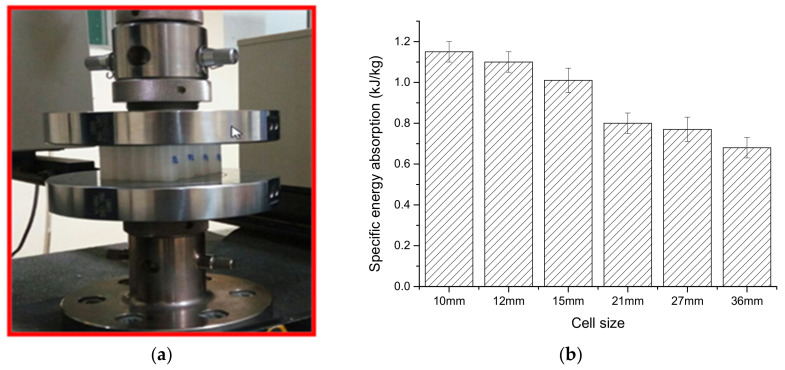
(**a**) Compression test setup; (**b**) Specific energy absorption vs. cell size [171].

**Figure 19:**
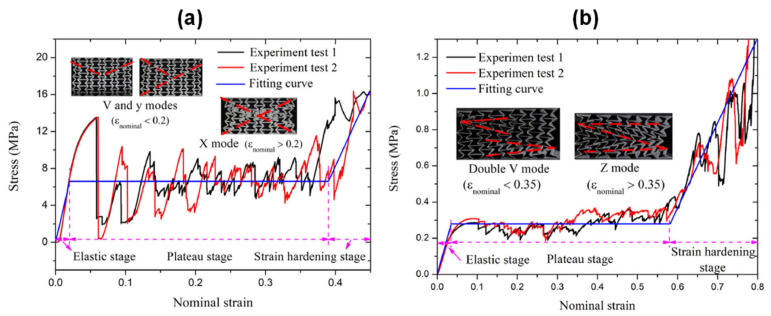
Stress vs. strain of (**a**) Thick-walled; (**b**) Thin-walled re-entrant honeycomb structure [173].

**Figure 20:**
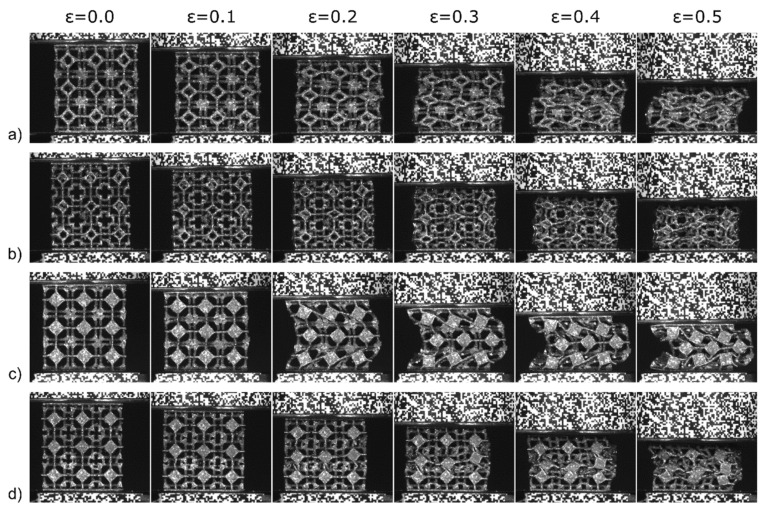
Deformation modes of (**a**) BD, (**b**) BS, (**c**) FD, (**d**) FS re-entrant tetra-kai-decahedral structure [174].

Sahu et al. [171] investigated the out-of-plane compressive behavior of the 3D printed honeycomb structure using a UTM experimental setup as shown in Figure 18a. The results obtained from the above test are shown in Figure 18b, where the graph between specific energy absorption (SEA) and cell size is drawn. It was noted that the lower cell size has the higher SEA due to the higher value of relative density. Ni et al. [172] investigated the compressive behaviour of open-cell copper foam with four types of geometrical constructions, i.e., strut, node, closed, and groove cell. It is observed that increasing the thickness of the strut increases the structural stability and the orientation of the strut which plays a major role in the load-bearing capability. Dong et al. [173] tested the compressive properties of two types of re-entrant honeycomb configurations, i.e., thick-walled (i.e., t ≥ 1 mm) and thin-walled (i.e., t < 1 mm) structures. Figure 19a,b shows the stress vs. strain of the thick-walled and thin-walled configurations, respectively, where it was evident from the result that during the first stage of the plateau region (i.e., ε_nominal_ < 0.2), the deformation was mainly due to the V and Y modes, and during the second stage (i.e., ε_nominal_ > 0.2), the deformation was only due to the X mode for the thick-walled configuration. However, the thin-walled configuration within the plateau stage showed insignificant influence on the crushing stress. Neuhauserova et al. [174] investigated the compressive properties of additively fabricated different re-entrant tetra-kai-decahedral structures, i.e., beam direct (BD), beam stem (BS), facet direct (FD), and facet stem (FS). It was noted that the BD structures exhibited the highest value of yield stress among all other tested samples. The deformation pattern is represented in Figure 20.

### 5.2. Three Point Bending Test

The three-point bending test is performed as per ASTM C 393 test standard [175]. The strength (σ_b_) and modulus during bending of the sandwich core beam is measured as per the following equation [176].

(3) $$\sigma_{b}=\frac{3PS}{2bd^{2}}$$

(4) $$E_{b}=\frac{S^{3}m}{4bd^{3}}$$

where, *P* = force at a given point; *S* = length of support span; *b* = width of the sandwich specimen; *d* = thickness of the sandwich specimen.

The energy absorption and specific energy absorption are obtained as per the following equation [176].

(5) $$EA=\int_{0}^{d}F\left(\delta \right)d\delta$$

(6) $$SEA=\frac{EA}{m}=\frac{\int_{0}^{d}F\left(\delta \right)d\delta }{m}$$

The symbols used above have similar meanings as in the literature [176].

**Figure 21:**
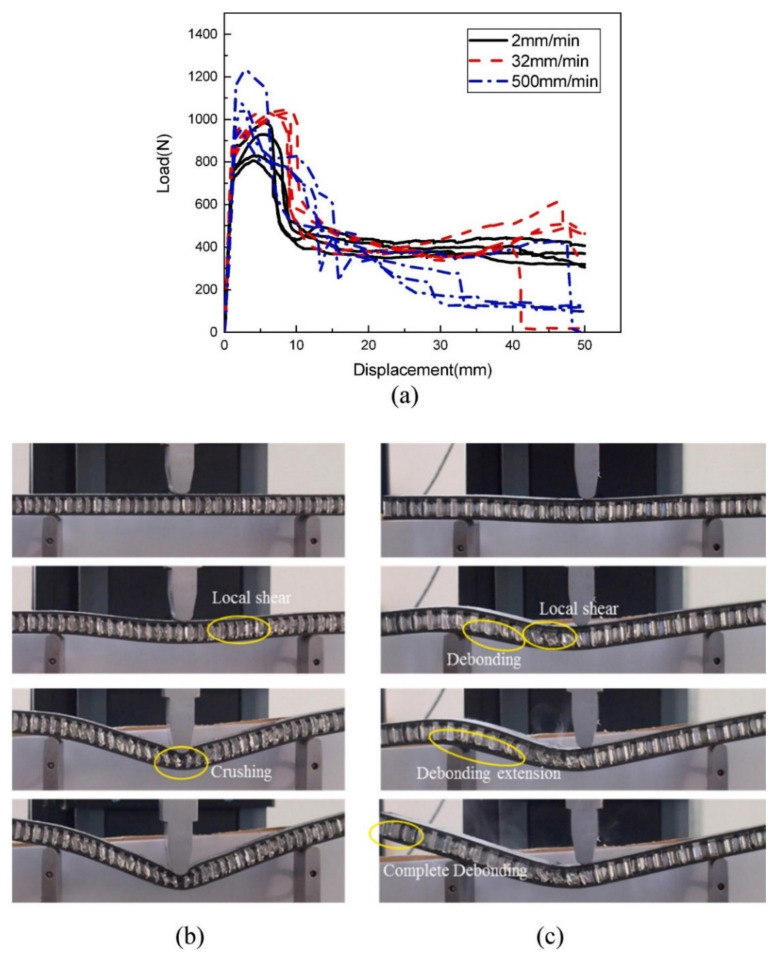
(**a**) Load displacement curve during three-point bending test; (**b**,**c**) Deformation mode of a sandwich specimen under three-point bending test [177].

Xiao et al. [176] reported the bending response of the aluminum honeycomb core with carbon fiber reinforced plastic (CFRP) under a quasi-static bending load. It was noted that the specific energy absorption (SEA) and energy absorption (EA) were greatly enhanced with the ±30° fiber direction. Sun et al. [177] carried out three-point bending analysis of aluminum honeycomb core with carbon fiber face sheet toughened by short aramid fiber tissues as well as carbon fiber belts. The cell size and wall thickness of the honeycomb were 6 mm and 0.06 mm, respectively. Figure 21a shows the load vs. displacement curve of three trials of sandwich core under three-point bending load. It was noted that the average peak load was increased by 39.8, 26.8, and 18.1% for interfacial toughening samples compared without toughening. The bending deformation behavior is represented in Figure 21b,c.

### 5.3. Impact Test

Impact testing is a crucial practice to measure the factors related to the dynamic fracture of composite sandwich material. The impact range is classified into low velocity and high velocity when the range is <10 m/s and >50 m/s, respectively [178]. The impact test of sandwich structure is normally carried out through a drop tower impact test equipment. The following important equation can be used to analyze the impact velocity [179].

Impact velocity

V = √2gh(7)

where, ‘g’ = acceleration due to gravity and ‘h’ = drop height in meter

Potential energy = mgh(8)

‘m’ = drop mass

**Figure 22:**
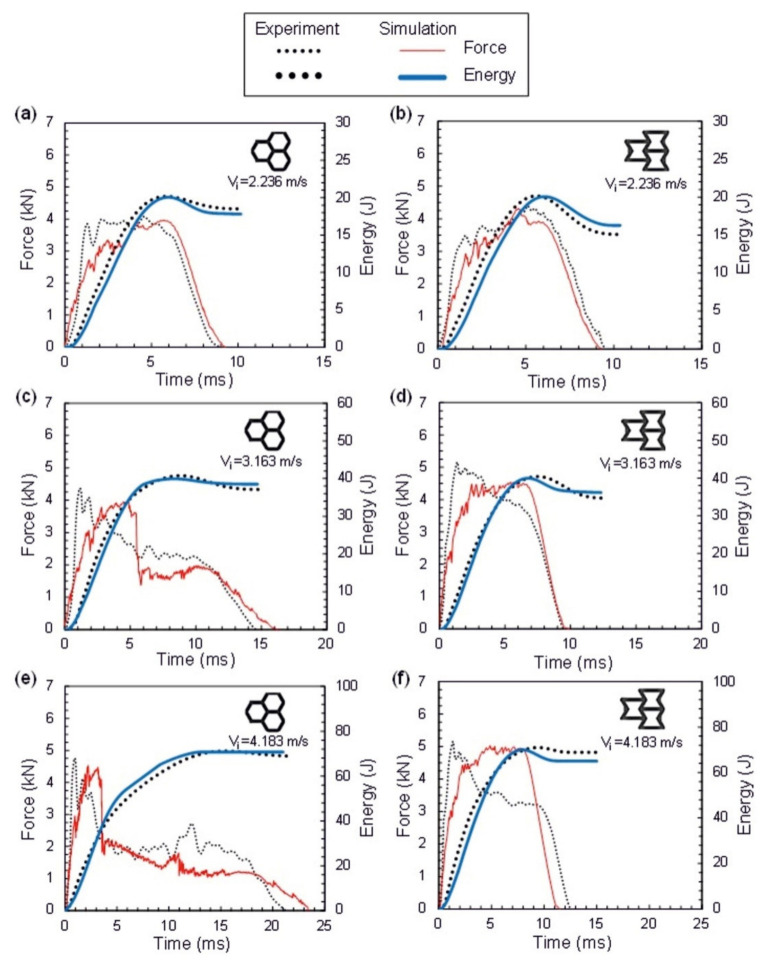
(**a**–**f**) Out-of-plane orientation experimental and FEM impact results of the honeycomb and re-entrant sandwich beam at velocities of 2.236, 3.163, and 4.183 m/s [180].

**Figure 23:**
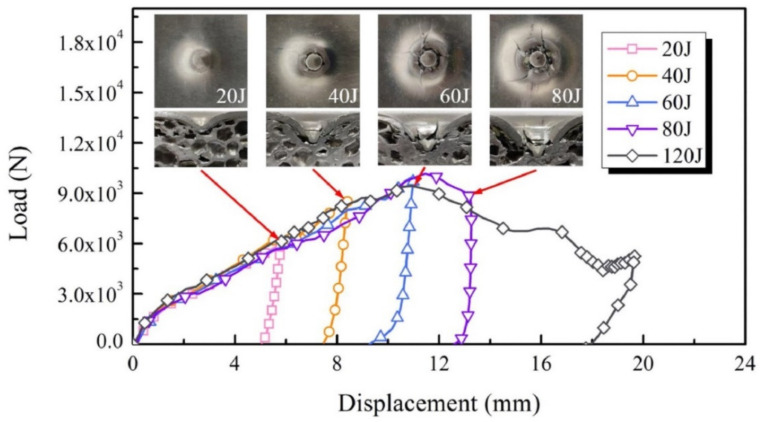
Impacting responses and the corresponding failure process of the sandwich panels using the conical impactor with different impacting energies [181].

Ozen et al. [180] reported the low velocity impact behaviour of acrylonitrile-butadiene-styrene (ABS) based thermoplastic re-entrant honeycomb cores and carbon fiber reinforced plastic (CFRP) face sheets at various impact energies, i.e., 20, 40, and 70 J. The specimens were fabricated via a 3D printing route and the test was carried out at both out-plane and in-plane orientations. Figure 22 illustrates the force vs. time vs. energy curve, and it was noted that the re-entrant honeycomb along the in-plane orientation revealed superior impact energy dissipation behavior when compared to out-of-plane orientations. Bates et al. [40] investigated drop weight impact analysis of the continuously graded structures (CGSs) and compared the results with uniform graded structures (UGSs). It was noted that the highest impact energy of 270 mJ/cm^3^ is noted for the UGSs. Huo et al. [181] performed impact analysis of sandwich structure filled with aluminum foam as core material and impacted with different shapes of impactor such as spherical, flat, and conical shapes. An infrequent deformation pattern is noted for the conical impactor as shown in Figure 23. The face sheet was fractured at an early stage and the impact resistance of the sandwich kept rising even though the face sheet was completely fractured.

## 6. Major Challenges

Sandwich structures made with creative core structures are used in a wide range of applications; however, there are several shortcomings, such as the heterogeneity and considerable mismatch in properties between core and face sheet, their fabrication, and the joining and mechanical testing which poses critical challenges [182]. Keeping in mind their application, there is a need for critical thinking in designing, selecting the material, and fabricating sandwich panels. The followings is the three major challenges that may be considered and are discussed.

### 6.1. Design Challenges

The optimum design of the composite structure has many applications in engineering problems. Reducing mass and increasing the stiffness are the key challenges faced during the designing of the core [183]. For the innovative core, obtaining arbitrary density is a unique feature, which can be exemplified as a design challenge. The optimal design of the core provides a unique challenge and opportunity to develop a new generation of sustainable and novel sandwich structures.

### 6.2. Material Challenges

The performance of the sandwich structure depends on the material selected for the fabrication of the skin and core. Sandwich structures made with metals and papers are usually adopted; however, most possess certain loopholes such as poor compliance during in-plane direction and the drawback of poor stiffness and low moisture resistance capability. If the core is fabricated with metallic material and at low density, it significantly reduces the stiffness as well as the strength characteristics [184]. The polymeric core material is supposed to be the best alternative to the above difficulty. The polymeric material opens up an extensive range of possibilities for customized sandwich fabrication with diverse properties [185,186].

### 6.3. Fabrication Challenges

The structural performance of the composite sandwich structure is significantly impacted by the fabrication route adopted. The conventional fabrication route, namely corrugation, expansion, and forming has challenges such as the higher cost of fabrication, inability to fabricate complex sandwich core designs, incompetence to handle mass production, an incapability with multiple materials. The additive manufacturing techniques (AMTs) may suitable to address the above issue, where the 3D part of the composite structure can be built with a high degree of accuracy directly from user-defined CAD data [187]. However, structural rigidity and warpage problems are the major setbacks here. Hence, maintaining suitable infill density and retaining a controlled environment are the key challenges.

## 7. Future Direction

Based on the extensive review work carried out, the future direction is suggested to undertake the work moving forward on the relevant area concerned.

- The use artificial intelligence/data mining and topology optimization to design the composite sandwich structure [188,189,190] for a specific application may be possibly carried out.
- Studies may also be performed to predict the damages on the crashworthiness or blast performance of composite sandwich structures and to propose suitable materials to reduce aging damage [191,192].
- The potential use of the innovative sandwich composite structure for the fabrication of the shape morphing for energy harvesting applications inspired by nature that activate with specific stimuli and retract back when the stimuli is removed is another emerging area of study [193].
- The fabrication of sandwich composites via natural fiber composites or bio-composites has potential use in the biomedical industries [194,195,196].
- The work towards structural health monitoring and optimization [197] of composite sandwich structures opens up new possibilities to explore. Vibro-acoustic analysis [198,199], viscoelastic analysis [200], and shielding structure analysis [201] are some prominent areas, which open new opportunities for innovative sandwich structures.
- Sandwich structures under impact damage can deteriorate the flexural properties of the composite by 50%, a good reason to localize skin buckling [202].
- Various self-healing materials may be implemented while fabricating composite sandwich structures to self-cure the composite when there is a damage [203].

## 8. Conclusions

The present investigation is a brief review of advanced sandwich structures. The design of the core, the material of the core, and the material for the skin, along with their joining method, play a deterministic role in achieving the fabrication of advanced sandwich beams. Sandwich structures normally have stiff facing sheets in addition to a lightweight core. The augmented load-bearing capabilities and the structural flexibility of sandwich beam conventional cores are modified with the folded and graded core. These traditional sandwich structures are best suitable for crashworthiness applications. However, innovative core materials such as shape memory alloy/polymer, piezoelectric, magneto-rheological fluid, as well as electrorheological fluid and elastomer sandwich beam may support the smart sandwich beam development. The smart sandwich structure has potential applications in areas such as micro-robotics, space applications; and marine applications.

## Figures and Tables

**Table 1:** Literature on FRP, MMC, and PMC.

Fibers	Chemical Treatment	Fabrication Technique	Reference
Jute fiber	Sodium hydroxide	Solution mixing	Orasugh et al. [138]
Flax fiber	Sodium hydroxide bleached with hydrogen peroxide	Solution casting	Mujtaba et al. [139]
Flax fabric	Sodium hydroxide, sodium carbonate	Solution casting	Csiszar et al. [140]
Kenaf fiber	Sodium hydroxide, sodium chlorite	Solution casting	Zainuddin et al. [141]
Nylon fiber mat	-	3D printing	Spackman et al. [142]
Pristine jute fiber	Sodium hydroxide, dimethyl sulfoxide	-	Lin et al. [143]
Copper	Graphene nanoplates	Spark plasma sintering	Shao et al. [144]
Ni	CNT	High-pressure torsion	Aristizabal et al. [145]
Copper	Diamond	Cold spray	Yin et al. [146]
Aluminum	Exfoliated graphite	Powder metallurgy	Alam et al. [147]
Stainless steel	Titanium carbonitrides	Sintering	Baken et al. [148]
HDPE	ND/CNT/GNP	Melt mixing	Sahu et al. [149]
Magnesium	CNT	Hot extrusion	Li et al. [150]
UHMWPE	CNT	Melt mixing	Yin et al. [151]
HDPE	Aluminum nitride	Melt blending	Rajeshwari et al. [152]
Epoxy	Kenaf	Hand lay-up technique	Saba et al. [153]
PP	GNP and CNT	Melt mixing and compression molding	Al-Saleh [154]
Epoxy	CNF and GF	Mechanical mixing and compression molding	Kavitha et al. [155]
Epoxy-Bisphenol A	Graphene and CNT	Mechanical mixing, molding, and curing	Shokrieh et al. [156]
UHMWPE	CNT	Melt mixing	Sreekanth et al. [157]

## Data Availability

Not applicable.
